# Anatomy-aware lymphoma lesion detection in whole-body PET/CT

**DOI:** 10.3389/fonc.2026.1695211

**Published:** 2026-05-22

**Authors:** Simone Bendazzoli, Antonios Tzortzakakis, Andréas Abrahamsson, Björn Engelbrekt Wahlin, Örjan Smedby, Maria Holstensson, Rodrigo Moreno

**Affiliations:** 1 Department of Biomedical Engineering and Health Systems, KTH, Royal Institute of Technology, Stockholm, Sweden; 2 Department of Clinical Sciences, Intervention and Engineering, Karolinska Institutet, Stockholm, Sweden; 3 Department of Nuclear Medicine and Medical Physics, Section Nuclear Medicine Huddinge, Karolinska University Hospital, Stockholm, Sweden; 4 Department of Nuclear Medicine and Medical Physics, Theranostics Trial Center, Karolinska University Hospital, Stockholm, Sweden; 5 Division of Hematology, Department of Medicine, Huddinge, Karolinska Institutet, Stockholm, Sweden; 6 Medical Unit Hematology, Solna, Cancer, Karolinska University Hospital, Stockholm, Sweden; 7 Division of Medical Radiation Physics, Department of Physics, Stockholm University, Stockholm, Sweden; 8 Department of Neurobiology, Care Sciences and Society, Karolinska Institutet, Stockholm, Sweden

**Keywords:** anatomical priors, lymphoma, medical object detection, nnDetection, PET/CT, Retina U-Net, Swin Transformer

## Abstract

**Motivation and objectives:**

Early cancer detection is essential for improving patient outcomes, and 18F-FDG PET/CT imaging plays a central role by combining metabolic and anatomical information. However, accurate lesion detection remains challenging due to the presence of multiple lesions with varying sizes and locations. This study investigates whether incorporating anatomical prior information can improve deep learning-based lesion detection performance.

**Methods:**

Anatomical priors were incorporated by adding organ segmentation masks generated with TotalSegmentator as auxiliary input channels to two lesion detection frameworks: the CNN-based nnDetection and a transformer-based Swin UNETR implemented in MONAI. The Swin Transformer was trained using a two-stage strategy, with self-supervised pretraining performed on the autoPET dataset and supervised fine-tuning of the detector model conducted on the independent Karolinska lymphoma dataset. Model evaluation followed a single hold-out split, and performance was assessed using FROC and average precision metrics.

**Results:**

Experiments were conducted on two independent PET/CT datasets covering different tracers and cancer subtypes. The autoPET dataset includes 18F-FDG PET/CT scans of lymphoma, melanoma, and lung cancer, while the Karolinska dataset focuses on lymphoma imaging. Incorporating anatomical priors consistently improved lesion detection performance within the nnDetection framework across both datasets. Specifically, nnDetection augmented with anatomical masks improved in mAP@0.1–0.5 from 0.288 to 0.335. In contrast, anatomical priors had minimal impact on the Swin Transformer, which did not demonstrate clear advantages over CNN-based encoders.

**Conclusions:**

Anatomy-aware priors substantially enhance lesion detection performance in CNN-based models, highlighting the importance of explicit anatomical context for multi-lesion PET/CT analysis. However, these benefits do not readily transfer to transformer-based architectures, indicating the need for improved strategies to integrate anatomical information into vision transformers for medical image analysis.

## 1 Introduction

Lymphoma is one of the most common hematological malignancies worldwide and includes a diverse group of diseases characterized by variable biological behavior, patterns of progression, and clinical outcomes. This heterogeneity contributes to differences in disease aggressiveness, treatment response, and prognosis across lymphoma subtypes (1, 2). Lymphoma often presents with multiple nodal and extranodal lesions scattered throughout the body, which complicates comprehensive evaluation. Accurately assessing all lesions, understanding their spatial distribution, and capturing the variability in disease behavior remain significant challenges in clinical imaging and analysis (3). For this reason, early detection of lymphoma is crucial for effective treatment and improves survival rates and patient quality of life by minimizing invasive procedures. Combined positron emission tomography (PET) and computed tomography (CT) (PET/CT) scans provide more comprehensive information about tumors, including anatomy and metabolism. Whole-body PET/CT detected new and unexpected primary malignant tumors in at least 1.2% of cancer patients (4), and PET/CT has demonstrated usefulness in early cancer diagnosis, potentially improving survival rates. Due to the characteristically elevated glucose metabolism of malignant lymphoid tissue, 18F-FDG PET/CT has become the standard imaging modality for most lymphoma subtypes, providing sensitive detection of both nodal and extranodal lesions and enabling detailed assessment of disease extent (5, 6).

In order to accurately identify and count multiple lesions in PET-CT, including smaller ones that might be overlooked by segmentation methods, automated methods for object detection have been proposed (7, 8). By prioritizing the identification of all potential lesions, regardless of size or location, object detection can contribute to more comprehensive and accurate cancer evaluations on PET/CT imaging, contributing to earlier and more accurate cancer detection, ultimately leading to better patient outcomes.

Object detection in PET/CT remains particularly challenging in multifocal disease, where the goal is to accurately identify and count all lesions, including the small ones that segmentation methods may miss. This capability is crucial in disseminated cancers, where comprehensive lesion detection directly impacts clinical assessment (8, 9).

Detection methods have demonstrated versatility across cancer types, proving effective for both localized tumors and disseminated diseases such as lymphoma and metastatic melanoma. They are especially well-suited for identifying organs and large lesions, as shown by frameworks like nnDetection (10) and Retina U-Net (11). In particular, nnDetection is considered the state-of-the-art for detection. This is a self-configuring framework for 2D and 3D medical object detection, designed to automatically adapt to new tasks and datasets. It has demonstrated competitive performance on several benchmarks, including the ADAM challenge for aneurysm detection and segmentation (12, 13) and the LUNA16 challenge for pulmonary nodule detection (14). The framework has also been applied effectively to detect intracranial aneurysms in TOF-MRA and structural MRI (15). nnDetection is built on the Retina U-Net architecture, which combines RetinaNet (16), a single-stage object detector network, with U-Net (17) to incorporate pixel-wise segmentation supervision, thus enhancing detection accuracy. This architecture has demonstrated strong performance in various clinical applications, including lung cancer staging using PET/CT (18) and kidney tumor detection in the KiTS21 dataset (19), consistently outperforming conventional object detection models in terms of average precision.

In recent years, the incorporation of anatomical context into medical images has gained attention as a means of integrating the information available to deep learning models. This additional context allows networks to infer higher-level representations during training, leading to a more comprehensive understanding of the task. Several studies have demonstrated the value of anatomical priors in deep learning applications. For example, Bermudez et al. (20) showed that integrating volumetric features of anatomical regions into a deep neural network for brain age prediction significantly enhanced performance compared to models trained solely on imaging data. Similarly, Jil et al. (21), reported improved accuracy in medical object detection and classification when anatomical guidance was explicitly provided during training. In the domain of segmentation, the In-context Cascade Segmentation (ICS) framework (22) achieved superior results in anatomically complex regions by enforcing anatomical consistency across image slices, outperforming baseline models.

More recently, the introduction of vision transformer (ViT)-based architectures into medical image analysis has led to notable advances in segmentation tasks. Among these, Swin UNeTR (23) replaces the traditional CNN encoder of U-Net (17) with a ViT (24), allowing the model to capture long-range spatial dependencies. The mechanisms from ViTs are similar to the way radiologists assess PET/CT scans, where they consider spatial patterns and co-occurrence of lesions across regions, rather than evaluating them in isolation. For example, the presence of a lesion in one area may suggest a possible involvement in another area through lymphatic spread, while the absence of lesions in certain regions may be a negative predictive factor for others. Thus, using this interregional context could significantly improve automated detection in such a complex problem. Thus, it is relevant to assess whether ViTs are appropriate for lesion detection tasks.

In this study, we explore training strategies to improve deep learning performance for lymphoma lesion detection on whole-body PET/CT scans, with a particular focus on integrating prior anatomical knowledge. Given the disseminated nature of lymphoma and the spatial complexity of whole-body imaging, our approach builds upon state-of-the-art methods while introducing architectural adaptations to better capture long-range relationships between lesions in anatomically distant regions. To incorporate anatomical context into the detection process, we developed an anatomy-aware framework that integrates segmentation masks of 104 organs, obtained from TotalSegmentator (25), directly into the training pipeline. This might help the model to better localize the lesions using structural information about the surrounding organs and tissues. Moreover, to explore whether ViT-based encoders can improve lesion detection, we replaced the standard CNN encoder of the Retina U-Net architecture (11) with a Swin Transformer (26), following a design similar to Swin UNeTR (23).

The code associated with this study can be accessed at the following links: https://github.com/minnelab/LymphomaDetection.git (Pipeline and Self-Supervised Learning source code) and https://github.com/minnelab/nnDetection.git.

## 2 Materials and methods

### 2.1 Datasets

In this study, we included two PET/CT datasets. The first, an open-access public dataset containing PET/CT scans with various annotated cancer lesions, was used for the self-supervised pretraining of the Swin Transformer. The second, a private collection acquired and annotated at Karolinska University Hospital, was used to train the detection model for the lymphoma lesion detection task.

#### 2.1.1 autoPET dataset

**Figure 1:**
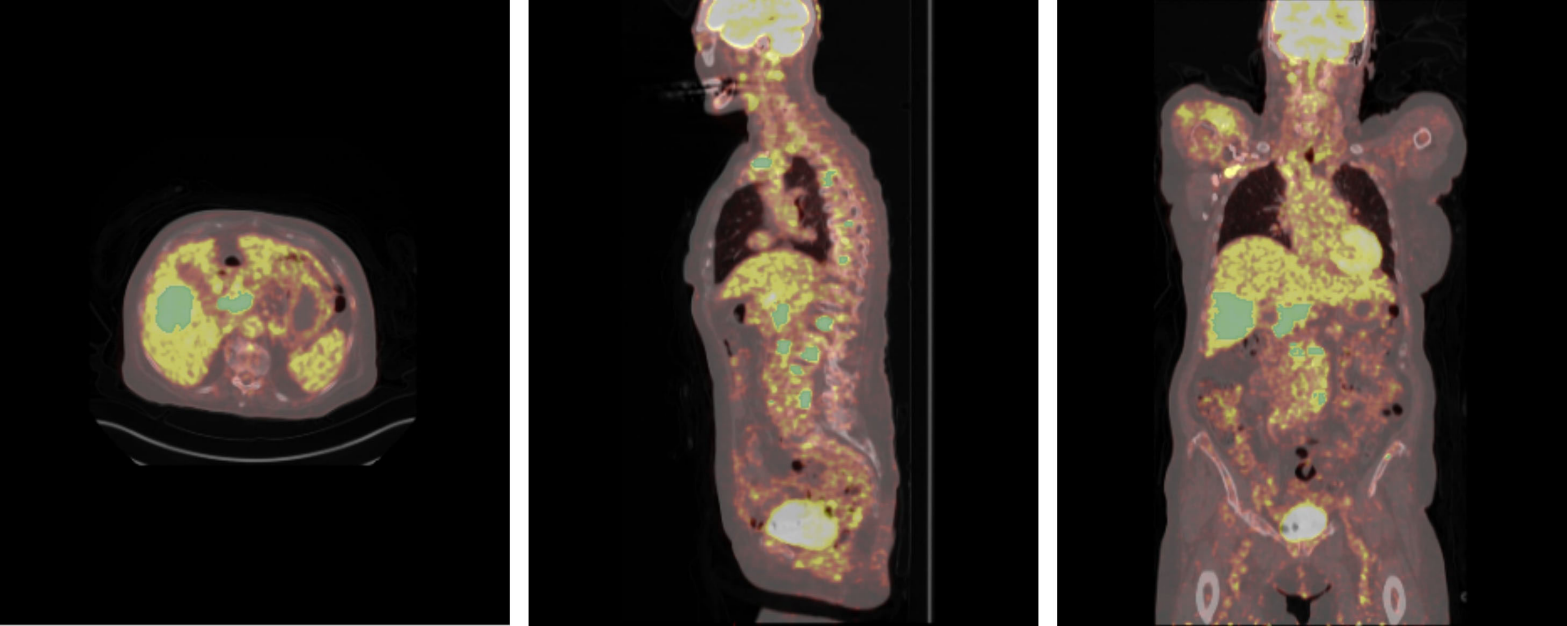
PET/CT scan from the AutoPET dataset, with manual annotations of lymphoma lesions highlighted in green. From left to right: axial, coronal, and sagittal views.

The first dataset used in this study is the autoPET dataset (27, 28). This dataset was used for the initial self-supervised pretraining stage of the Swin Transformer in our framework. The dataset comprises 1,611 whole-body PET/CT scans (an example is shown in **Figure 1**). These include data from patients with histologically confirmed malignant melanoma (*n* = 192), lymphoma (*n* = 155), prostate carcinoma (*n* = 332), or lung cancer (*n* = 193), as well as a set of negative controls (*n* = 739). PET acquisitions were performed using three different tracers: 597 prostate-specific membrane antigen ([^18^F]PSMA (*n* = 369) or [^68^Ga]PSMA (*n* = 228)) scans and 1,014 fluorodeoxyglucose ([^18^F]FDG). The subtype of lymphomas is not available for this dataset.

The inclusion of diverse cancer types was motivated by the requirements of our self-supervised pretraining strategy. Since this approach focuses on learning generalizable feature representations from the intrinsic structure of PET/CT data without relying on ground-truth labels, maximizing both data volume and diversity is more advantageous than restricting the cohort to a single pathology. This broad pretraining enables the model to capture a robust imaging representation that provides a powerful foundation for the subsequent, specialized lymphoma detection task.

Given the focus of this study on lymphoma lesion detection, we selected only the 872 PET/CT volumes with annotated cancer lesions for further use. Specifically, images from patients with lung cancer and melanoma, as well as prostate cancer, were included, given the specific purpose of this dataset to provide self-supervised information on how to extract lesion features from PET/CT. Each case includes a 3D whole-body PET volume, a corresponding 3D whole-body CT scan, and a 3D binary mask of manually segmented tumor lesions. The FDG PET/CT data were annotated by a radiologist experienced in hybrid imaging, while the PSMA PET/CT data were annotated by a single reader and subsequently reviewed by a radiologist with expertise in hybrid imaging. PET and CT data were acquired sequentially on state-of-the-art hybrid PET/CT scanners (Siemens Biograph mCT for FDG-PET/CT, Siemens Biograph 64-4R TruePoint, Siemens Biograph mCT Flow 20, and GE Discovery 690 for PSMA-PET/CT) at two sites. The use of hybrid imaging ensures anatomical alignment, with only minor discrepancies potentially arising from physiological motion.

The scan range typically extended from the skull base to the mid-thigh, although in clinically indicated cases, whole-body coverage was performed. For FDG-PET, an average dose of approximately 350 MBq of ^18^F-FDG was administered intravenously, with imaging initiated approximately 60 min post-injection. For PSMA-PET, imaging commenced on average 74 min after intravenous administration of 246 MBq ^18^F-PSMA (mean, 369 studies) or 214 MBq ^68^Ga-PSMA.

Diagnostic contrast-enhanced CT scans were subsequently acquired, covering the neck, thorax, abdomen, and pelvis. The CT slice thickness corresponding to FDG-PET was 2–3 mm. For PSMA-PET, CT slice thickness was 3 mm for the Siemens Biograph 64 and Biograph mCT systems and 2.5 mm for the GE Discovery 690.

#### 2.1.2 Indolent lymphoma KUH dataset

We also incorporated a second PET/CT dataset from Karolinska University Hospital (KUH); eligibility required a biopsy-confirmed diagnosis of lymphoma, across various subtypes and stages. This dataset was used in the second stage of our pipeline, focusing on training and evaluating the lymphoma lesion detection model.

The annotation process involved two distinct approaches: one radiologist manually annotated 77 cases using a 2D brush tool—identifying suspected lesions via high PET uptake and anatomical confirmation on CT—while a second radiologist annotated the remaining 151 cases using a semi-automatic method. In the latter, an automated PET-based algorithm first identified candidate high-uptake regions, which were subsequently refined by the expert. This heterogeneity in annotation protocols, involving different experts and a mix of manual and assisted tools, closely mirrors real-world clinical environments. By incorporating these variations during training, the resulting model gains robustness against annotation variability, enhancing its reliability for deployment across diverse institutions. All patients had biopsy-confirmed lymphoma, and lesion boundaries were defined based on expert interpretation of the PET/CT images. PET and CT volumes were acquired on hybrid state-of-the-art PET/CT scanners (Siemens Biograph 128, Siemens Somatom Definition AS, GE Medical Systems Discovery) from two different sites.

The scan range typically extended from the skull base to the mid-thigh, although whole-body coverage was performed in clinically relevant cases. Patients received an intravenous injection of on average 230 MBq of ^18^F-FDG, and PET acquisition was initiated approximately 70 min after injection.

For this study, attenuation correction CT (ACCT) was considered the primary modality of CT under investigation, since only 23 studies included diagnostic CT. For the Siemens Biograph and Siemens SOMATOM scanners, the CT slice thickness was 5 mm, while for the GE Discovery system, it was 3.75 mm.

All lymphoma diagnoses were confirmed by histopathological biopsy. For each patient, multiple imaging studies were available, covering primary diagnosis and subsequent follow-up examinations, allowing longitudinal assessment of disease progression and treatment response, including evaluation of complete response. Lymphomas were categorized into indolent and high-grade entities based on their biological behavior and clinical aggressiveness. Indolent lymphomas included follicular lymphoma grades 1–3A (FL), chronic lymphocytic leukemia (KLL), and marginal zone lymphoma (MZL). These entities typically exhibit slow disease progression but may undergo histological transformation into more aggressive forms. High-grade lymphomas comprised transformed follicular lymphoma (tFL), diffuse large B-cell lymphoma (DLBCL), and follicular lymphoma grade 3B (FL3B), which are characterized by rapid progression and require intensive treatment. In this cohort, transformation from indolent to aggressive lymphoma was explicitly represented by cases of tFL (see **Table 1**). In 151 cases, we did not have the subtype of lymphoma.

**Table 1:** Patient cohort and lymphoma diagnosis distribution for the KUH dataset.

Category	Diagnosis	Patients	Studies
*Indolent*	Follicular lymphoma (FL) Chronic lymphocytic leukemia (KLL) Marginal zone lymphoma (MZL)	32 2 2	60 2 3
*High-grade*	Transformed follicular lymphoma (tFL) Diffuse large B-cell lymphoma (DLBCL)	4 1	7 3
	Follicular lymphoma grade 3B (FL3B)	2	2
*Non-available*		151	151
Total		194	228

### 2.2 Deep learning framework and network architectures

On both self-supervised and object detection fine-tuning, an 80:20 patient-level split hold-out train test was adopted to evaluate performances on an independent test set.

#### 2.2.1 nnDetection framework

For the object detection task, we built on nnDetection (10). nnDetection is the state-of-the-art for object detection in medical imaging. Built on the same principles as *nnU-Net* (29), nnDetection is a self-configuring framework specifically designed for the detection of medical objects. The framework is designed to automatically select a range of hyperparameters, training configurations, and validation strategies through rule-based heuristics, based on the characteristics of the dataset under investigation and the objects of interest, in our case, lymphoma lesions. nnDetection generates that way a fingerprint of the dataset that is then used to optimize the architecture and hyperparameters so that the neural network is optimal for the task at hand.

In our case, the fingerprint of the dataset under analysis with nnDetection includes the spacing, shape, modality, intensity distribution, and number and size of the objects. Based on this fingerprint, several parameters are tuned to optimize the training and inference processes. These include the choice between full-resolution or cascade training (i.e., low-resolution followed by high-resolution stages), the batch size, and the patch size, as well as the resampling and normalization strategies used during data preprocessing.

The nnDetection framework adopts the Retina U-Net architecture. Retina U-Net is a one-stage object detection model, structurally similar to RetinaNet (16) and specifically designed to address challenges such as class imbalance and multiscale object detection in medical imaging. nnDetection uses the fingerprint of the dataset to optimize the architecture. Additional parameters are tuned during the empirical optimization performed in the validation phase, including the non-maximum suppression (NMS) threshold, the minimum object size (to filter out small detections), the intersection over union (IoU) threshold, and the minimum model confidence score required to classify prediction boxes.

Retina U-Net utilizes anchor boxes, predefined bounding boxes with varying sizes and aspect ratios, to detect objects at different locations and scales. A significant innovation introduced in RetinaNet and also incorporated in Retina U-Net is the focal loss. This loss function is specifically designed to tackle class imbalance by placing greater emphasis on hard-to-classify examples, achieved by focusing training on hard negative boxes, which are those that the model fails to detect. The backbone network, typically a convolutional neural network, in this case a U-Net encoder, serves as the foundation for extracting feature maps from the input images. Subsequently, these feature maps are processed by a feature pyramid network (FPN), which generates a multiscale feature pyramid. The FPN integrates a bottom-up pathway, which involves upsampling coarser feature maps, alongside lateral connections that merge these upsampled maps with matching finer feature maps from the encoder.

Two subnets operate on the FPN’s output at each stage, excluding the highest resolution one: a *classification head* and a *regression head*. The classification head predicts the probability that an object is present at each spatial location for predefined anchor boxes and object classes, while the regression head refines the bounding box coordinates by predicting a center offset and shape ratio for each predefined anchor box. Both subnets use convolutional layers to process the feature maps produced by the FPN. A key difference between Retina U-Net and RetinaNet is that the highest resolution stage of the FPN in Retina U-Net is also used for an auxiliary segmentation task. According to the original paper, the gradient flow from this auxiliary task during backpropagation benefits the lower resolution stages involved in the object detection task.

#### 2.2.2 Inclusion of anatomical information

**Figure 2:**
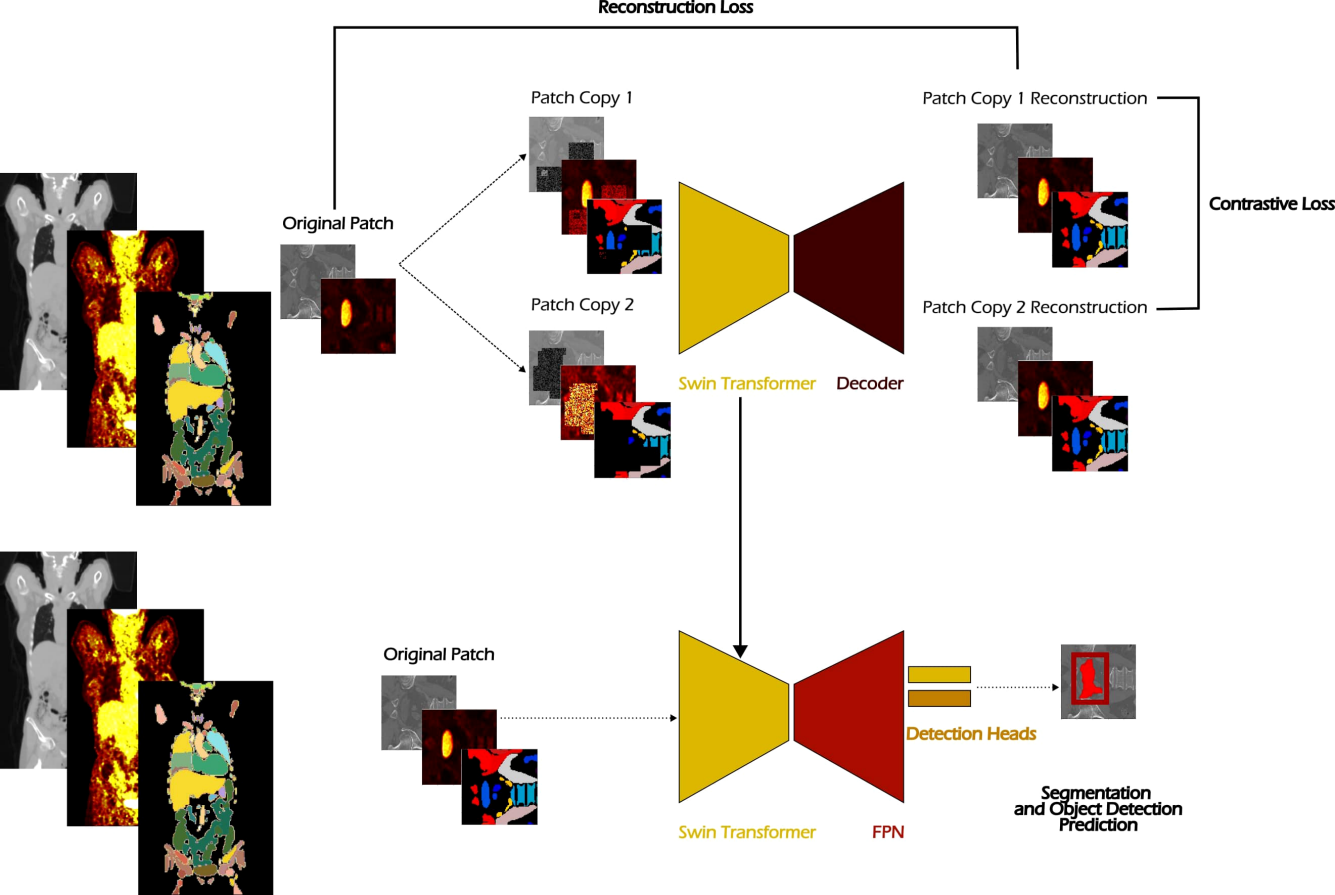
Overview of the experimental workflow integrating anatomical information via TotalSegmentator predictions. The generated segmentation masks are utilized in both the self-supervised pretraining phase, where they are included as input alongside corrupted PET/CT patches, and in the subsequent object detection training phase, where they are provided as additional input channels to the network.

To assess the impact of incorporating anatomical information as prior knowledge, the experimental procedure described above was performed in two variants. In the first variant, only PET and CT volumes were provided as input to the network, both during the self-supervised pretraining and the subsequent object detection training phase (see **Figure 2**). In the second variant, a third input channel was added, consisting of anatomical segmentation masks generated by the *TotalSegmentator* tool. These masks, which cover 104 anatomical structures, were obtained by performing inference on the CT modality alone and served as an additional source of anatomical context. Crucially, to ensure a fair comparison and eliminate any bias from previous configurations, the nnDetection framework was re-executed entirely from scratch for each variant. This involved a complete re-computation of the dataset fingerprints, preprocessing parameters, and training configurations.

#### 2.2.3 Self-supervised pretraining of Swin Transformer

ViTs, including the Swin Transformer, are typically high-parameter models that are computationally intensive to train. To mitigate these demands, the Swin Transformer replaces the global self-attention of vanilla ViTs with a hierarchical, window-based architecture. This design enhances computational efficiency and accuracy in dense prediction tasks, where capturing both fine-grained local detail and broad multiscale context is critical (30). By pairing this hierarchical 3D encoder with a U-Net-like decoder, Swin UNETR has achieved state-of-the-art Dice scores on benchmarks such as BTCV and the Medical Segmentation Decathlon, consistently outperforming ViT-based UNETR and other transformer variants in 3D medical segmentation (31).

To further optimize these large-scale architectures, a common strategy is to apply self-supervised pretraining to the transformer component, effectively “warming up” the model’s weights before supervised fine-tuning. This approach was notably implemented in (31), where Swin UNETR was pretrained using specialized proxy tasks. Crucially, the authors’ ablation study demonstrates that this self-supervised initialization is vital for learning robust volumetric representations and reaching peak performance. Such evidence reinforces the rationale for adopting pretraining strategies to improve convergence and accuracy, particularly in data-constrained medical domains. This methodology is further supported by the MoBY framework (32), which adapts contrastive learning techniques like MoCo-v2 (33) and BYOL (34) for Swin Transformers, showing strong transferability across diverse downstream detection and segmentation tasks.

Thus, for this initial self-supervised stage, we used the MONAI framework (35), which offers a rich collection of tutorials and modular components designed for deep learning in medical imaging. It includes numerous community-driven implementations of state-of-the-art architectures, image processing, and augmentation transforms, as well as utilities for training and validation.

**Figure 3:**
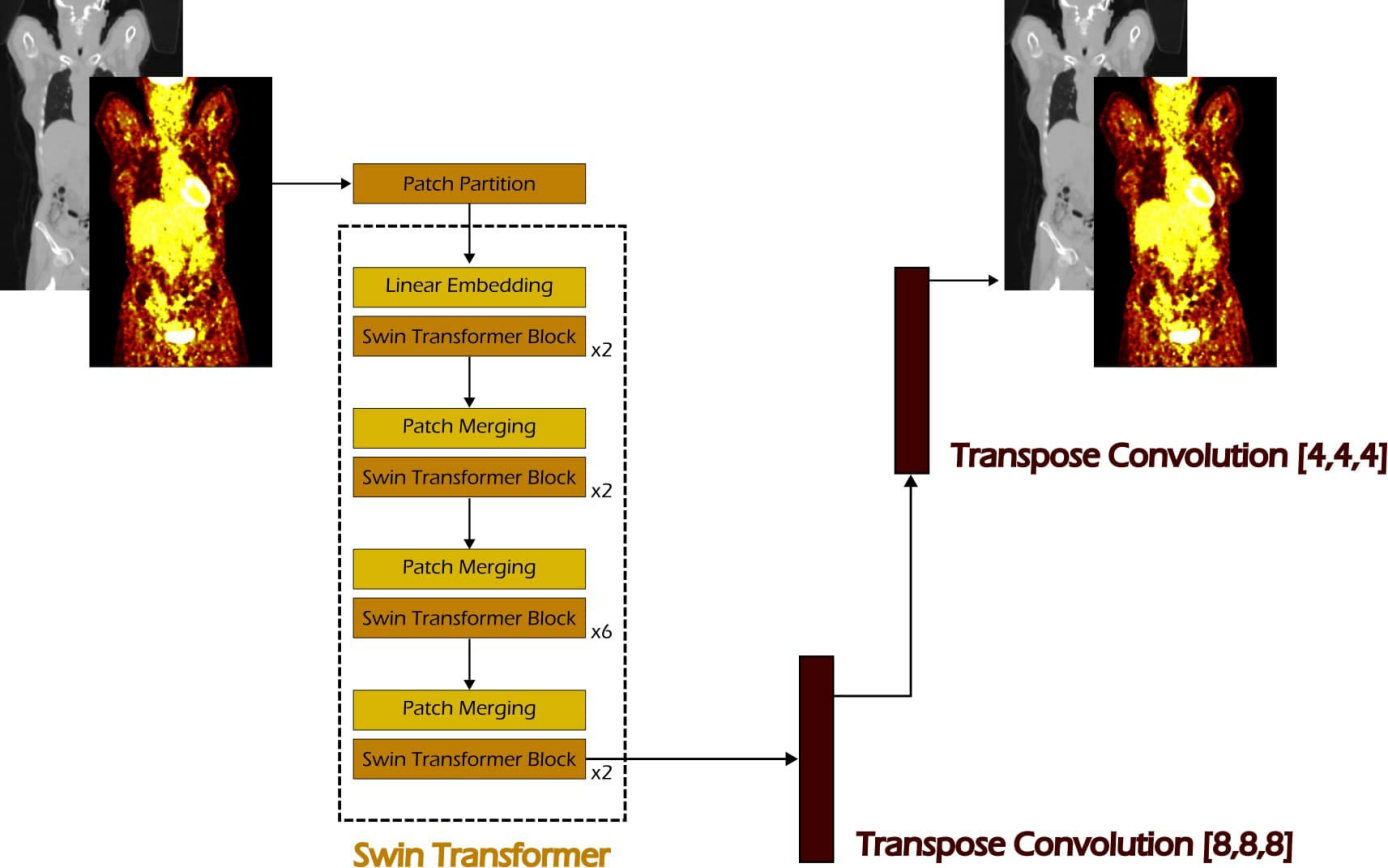
The proposed Swin Autoencoder network for the self-supervised task. A Swin Transformer is used as the feature extractor for the input PET/CT volumes, followed by a sequence of two transposed convolution layers to upsample the extracted features back to the original image resolution.

As shown in **Figure 3**, building on MONAI’s implementation of the Swin Transformer and drawing inspiration from self-supervised learning strategies used with ViT (24), we extended the architecture by connecting the Swin Transformer bottleneck to a decoder composed of two transposed convolution layers, forming an autoencoder (see **Figure 3**). This design enabled the model to be trained on a self-supervised image reconstruction task. The Swin Transformer used in our setup consists of four stages, with the number of Swin Transformer blocks set to 2, 2, 6, and 2, respectively. The embedding dimension *C* is set to 96, corresponding to the Swin-T architecture as defined in the original Swin Transformer paper (26). The window size is set to 4 × 4 × 4, and the number of attention heads for each stage is 3, 6, 12, and 24, respectively.

The learning objective was to reconstruct corrupted input volumes, inspired by the masked autoencoding strategy described in (31). A randomly cropped 3D patch from the input volume is duplicated, and each of the two copies is independently corrupted using a combination of the following transformations:

- Coarse dropout: Random rectangular regions were replaced with a random value in the range [0,0.2] or retained while the remaining areas of the image were filled with random values in the range [0,0.2], as described in (36, 37).
- Pixel shuffling: Random regions within the image were selected, and the pixels within each region were shuffled independently per channel (38).

The reconstruction task was optimized using a combined loss function composed of a reconstruction loss term ( ${ℒ}_{\text{rec}}$) and a contrastive loss term ( ${ℒ}_{\text{con}}$) based on (39), with a temperature parameter of 0.05. In this formulation, the contrastive loss is scaled by the reconstruction loss value, such that the total loss is defined as in Equation 1:

(1) $${ℒ}_{\text{total}}={ℒ}_{\text{rec}}+{ℒ}_{\text{con}}\cdot {ℒ}_{\text{rec}}$$

Training was performed for 500 epochs using the Adam optimizer with a fixed learning rate of 1 × 10^−4^. The input volumes were randomly cropped into patches of size 96 × 96 × 96, and a batch size of 2 was used.

From the available 872 PET/CT volumes with annotated cancer lesions in the autoPET dataset, 698 were used for training and 174 for validation in a single train–validation split.

#### 2.2.4 Swin RetinaUNeTR

**Figure 4:**
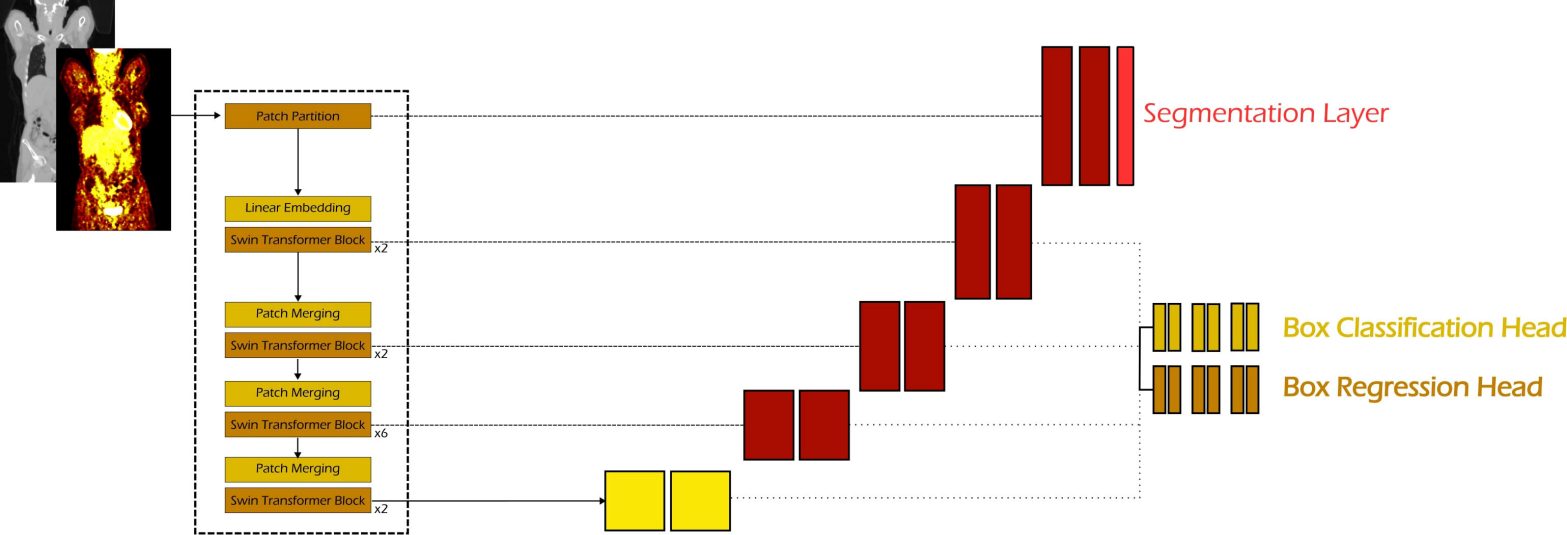
Proposed Swin RetinaUNeTR architecture. While maintaining structural similarity to Retina U-Net in the decoder and detection head components, the convolutional encoder is replaced by a Swin Transformer, serving as a vision transformer-based feature extractor.

Based on the two primary architectures, Retina U-Net and the Swin Transformer, we assess the performance of ViT-based encoders on feature extraction for lymphoma lesion detection. In this model, the U-Net encoder of Retina U-Net is replaced by a Swin Transformer, as illustrated in **Figure 4**. The integration is achieved through a hierarchical mapping where the output of each Swin Transformer stage is coupled with a corresponding level in the FPN. Specifically, the feature maps *S_i_* from the four stages of the Swin block, operating at downsampling ratios of 2, 4, 8, 16 relative to the input, are extracted and fed into the FPN via lateral connections. This ensures that the FPN receives a multiscale representation where the high-resolution, fine-grained details from the early layers and the low-resolution, semantically dense features from the deeper layers are aligned at each pyramid scale. By replacing the standard convolutional backbone with this transformer-based hierarchy, the FPN inherits long-range spatial dependencies and global context at every scale of the detection neck.

Thus, following the self-supervised pretraining phase, the Swin Transformer encoder was integrated with an FPN and detection heads within the nnDetection framework to train the Swin RetinaUneTR architecture for the lymphoma lesion detection task (see **Figure 4**).

Training hyperparameters were automatically selected by nnDetection, based on a fingerprint extracted from the input dataset. The multitask objective involved a combination of loss functions (Equation 2): Dice and cross-entropy losses for the segmentation component, Generalized Intersection over Union (GIoU) loss for bounding box regression, and standard cross-entropy loss for bounding box classification:

(2) $${ℒ}_{\text{total}}=\underset{\text{Segmentation}}{\underset{︸}{{ℒ}_{\text{Dice}}^{\text{seg}}+{ℒ}_{\text{CE}}^{\text{seg}}}}+\underset{\text{Box Regression}}{\underset{︸}{{ℒ}_{\text{GIoU}}^{\text{reg}}}}+\underset{\text{Box Classification}}{\underset{︸}{{ℒ}_{\text{CE}}^{\text{cls}}}}$$

Model training was performed for 50 epochs, followed by an additional epoch of stochastic weight averaging (SWA) (40). Each epoch consisted of 2,500 iterations, following the default nnDetection hyperparameter configuration, during which the training samples could be traversed multiple times if necessary, using a batch size of 2 and a patch size of 96 × 96 × 96 voxels.

The data augmentation strategy included a rich set of geometric and intensity-based transformations. Geometric augmentations comprised random elastic deformations, 3D rotations along all spatial axes, isotropic or anisotropic scaling, and random mirror flips across specified axes. Intensity enhancements included the application of Gaussian noise (10% probability), Gaussian blurring with varying sigma values (applied to 20% of samples), brightness scaling (multiplicative range [0.75, 1.25] with 15% probability), and optional additive brightness perturbations sampled from a Gaussian distribution. Further intensity manipulations included contrast adjustment and gamma correction, encompassing both standard and inverted gamma transformations, with configurable per-sample probabilities.

Stochastic gradient descent (SGD) was used as optimizer, with an initial learning rate of 0.01, momentum of 0.9, Nesterov acceleration enabled, and a weight decay factor of 3 × 10^−5^. The scheduling of the learning rate followed a warm polynomial decay strategy: the warm phase lasted 4,000 iterations starting from an initial learning rate of 1 × 10^−6^, followed by a polynomial decay with a gamma parameter of 0.9.

### 2.3 Metrics

For the object detection task, model performance was evaluated using average precision (AP) and the free-response receiver operating characteristic (FROC) score, in accordance with the recommendations of the *Metrics Reloaded* framework (41). Both metrics are well established in medical imaging for lesion detection and localization and belong to the family of multithreshold evaluation metrics, enabling performance assessment across varying decision thresholds.

AP summarizes the precision–recall relationship into a single scalar value and quantifies the ability of the model to correctly detect lesions while limiting false positives across confidence thresholds. Given a ranked list of detections, AP is computed as the discrete sum of precision values at increasing recall levels, as presented in Equation 3:

(3) $$\text{AP}=\sum_{n=1}^{N}\left(R_{n}-R_{n-1}\right)P_{n},$$

where *P_n_* and *R_n_* denote the precision and recall at the *n*-th threshold, respectively, and *N* is the total number of evaluated thresholds.

FROC analysis was used to assess lesion-level detection performance while explicitly accounting for the number of false positives per scan, which is particularly important in whole-body PET/CT studies where multiple lesions may be present. The FROC curve plots sensitivity as a function of the average number of false positives per image. Sensitivity is defined in Equation 4:

(4) $$\text{Sensitivity}=\frac{TP}{TP+FN},$$

where TP and FN denote the number of true positive and false negative lesions, respectively.

## 3 Results

This section presents the experimental results obtained in this study. We first report the performance of the proposed self-supervised learning task for the Swin Transformer, followed by a detailed analysis of the results for the object detection task on the PET/CT lymphoma dataset. Because object detection performance was evaluated on a single 80:20 held-out split, the results represent single scores for each metric. Consequently, formal statistical comparisons across methods were not performed, as the single-split approach does not provide the variance necessary for significance testing.

**Figure 5:**
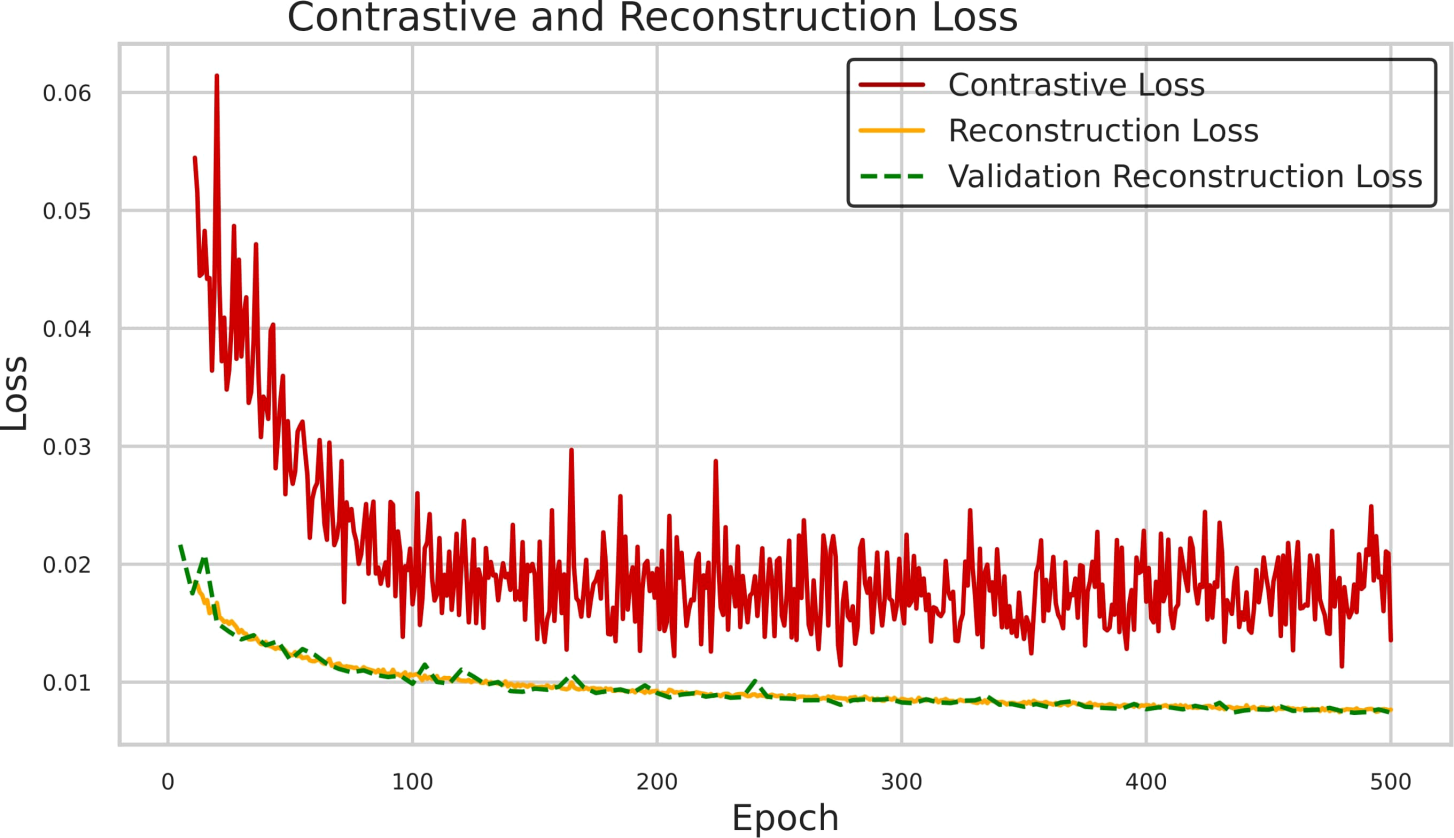
Training and validation loss curves across epochs. The training loss is split into contrastive and reconstruction components, shown separately. For validation, only the reconstruction loss is computed and displayed.

**Figure 6:**
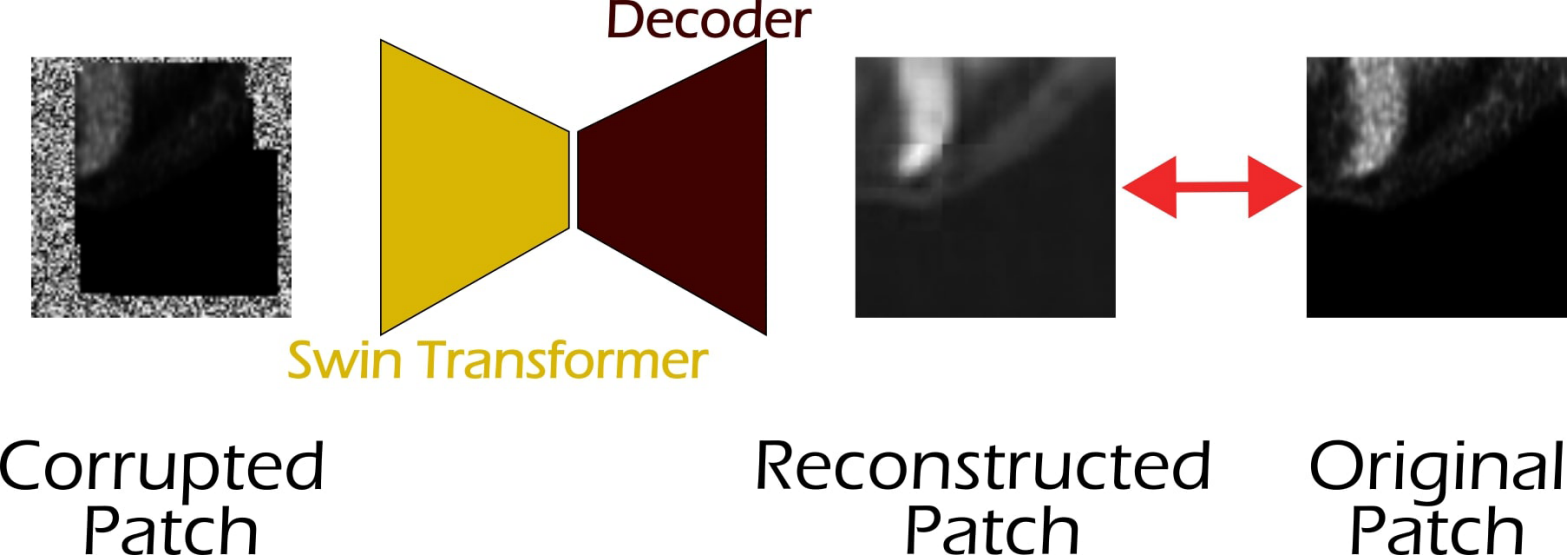
A randomly cropped 96 × 96 × 96 sample from the validation set, corrupted during augmentation and subsequently reconstructed by the Swin autoencoder network.

As illustrated in **Figure 5**, the training and validation curves for the self-supervised learning task indicate a stable and satisfactory convergence. The autoencoder effectively learned the reconstruction objective, as evidenced by the consistent decrease in both the reconstruction loss (*L1 loss*) and the contrastive loss during training. For the validation set, only the reconstruction loss is shown. Furthermore, **Figure 6** presents a representative example of the validation set. The input patch, sized 96 × 96 × 96, was corrupted using random pixel shuffling and coarse dropout. The autoencoder reconstruction is compared with the original uncorrupted patch, demonstrating the model’s ability to recover a meaningful structure from perturbed input data.

TS refers to the inclusion of TotalSegmentator masks as additional input channels. Metrics reported are FROC at IoU thresholds 0.1 and 0.5, AP at IoU 0.1 and 0.5, and mean AP (mAP) averaged over IoUs from 0.1 to 0.5.

**Table 2** summarizes the performance of four experimental setups evaluated using key object detection metrics: FROC@0.1, AP@0.1, and mAP. Among them, the nnDetection + TS model, where TotalSegmentator anatomical masks are added as input channels, achieves the best results in all three metrics, with a FROC@0.1 of 0.343, AP@0.1 of 0.499, and mAP of 0.335. These values represent relative.

**Table 2:** Object detection performance across all experimental setups.

Experiment	FROC@0.1	FROC@0.5	AP@0.1	AP@0.5	mAP@0.1–0.5
nnDetection [baseline]	0.284	0.115	0.418	0.110	0.288
nnDetection + TS	**0.343**	**0.130**	**0.499**	**0.129**	**0.335**
Swin RetinaUNeTR	0.243	0.074	0.372	0.065	0.237
Swin RetinaUNeTR + TS	0.244	0.077	0.376	0.070	0.234

Top scores for each metric are presented in bold.

**Figure 7:**
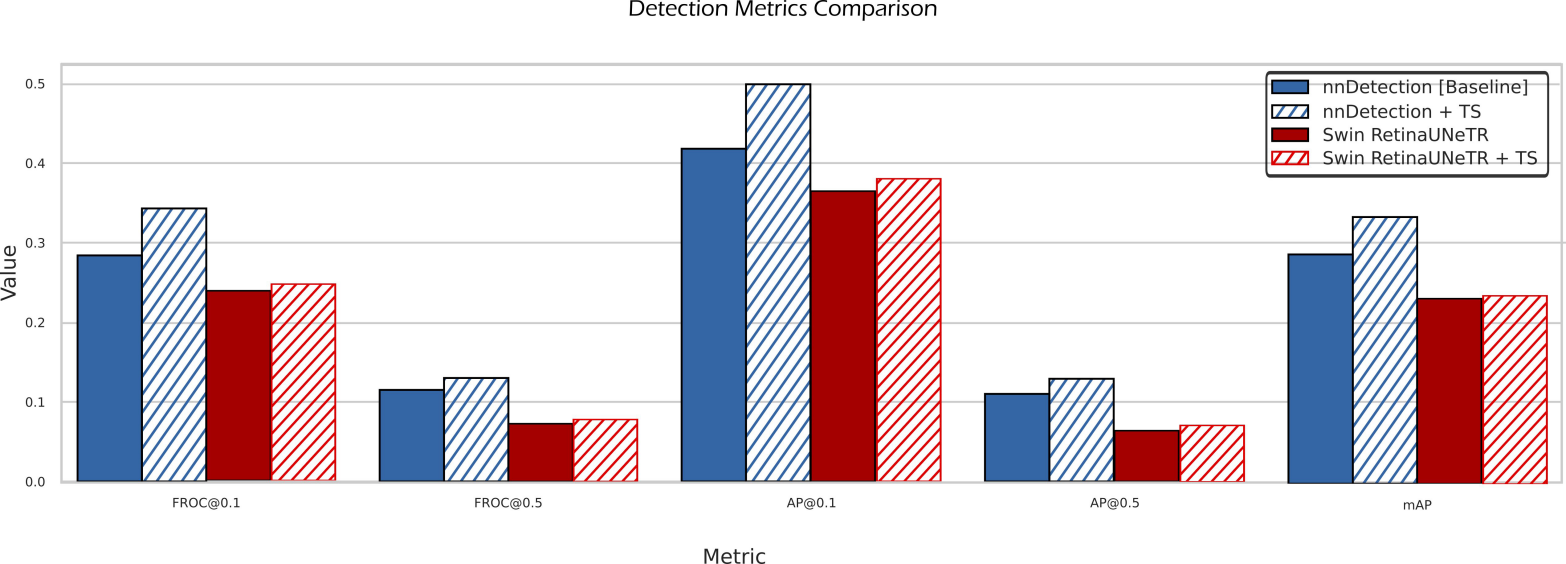
Comparison of detection metrics between the baseline nnDetection model, the Swin RetinaUNeTR model, and their enhanced versions that incorporate TotalSegmentator masks as additional input. Shown metrics include FROC@0.1, FROC@0.5, AP@0.1, AP@0.5, and mAP.

The annotator number is shown in brackets. gains of 0.059, 0.081, and 0.047, respectively, over the nnDetection baseline (FROC@0.1 = 0.284, AP@0.1 = 0.418, mAP = 0.288), as shown in **Figure 7**.

In contrast, as illustrated in **Figure 7**, adding the same anatomical information to the Swin RetinaUNeTR model produces only marginal gains, resulting in 0.244 (FROC@0.1), 0.376 (AP@0.1), and 0.234 (mAP), which are nearly identical to the baseline performance of Swin RetinaUNeTR. Despite the present improvements, the performance metrics remain relatively low in absolute terms, highlighting the significant complexity and inherent challenges of applying deep learning models to this specific detection task.

When directly comparing the Swin RetinaUNeTR model to the nnDetection baseline (see **Figure 7**), the latter clearly demonstrates superior performance in all three detection metrics. The nnDetection baseline surpasses Swin RetinaUNeTR with a FROC@0.1 of 0.284 vs. 0.243, an AP@0.1 of 0.418 vs. 0.372, and a mAP of 0.288 vs. 0.237. These results indicate that nnDetection offers more robust low-threshold sensitivity and precision, as well as better overall localization performance across the IoU range.

Finally, we report detection performance stratified by annotator in **Table 3**, providing a clear representation of how the model performs across the distinct annotation protocols utilized in this study. As shown, performance is consistently higher for Annotator 2; this highlights the importance of including heterogeneous data and accounting for variations in annotation protocols, which more accurately reflects the diversity of real-world clinical scenarios. In summary, integrating anatomical priors through TotalSegmentator masks consistently enhances the performance of the nnDetection framework, making it the most effective strategy among all tested configurations. In contrast, the Swin RetinaUNeTR architecture shows minimal responsiveness to the same anatomical input, suggesting that its ViT-based design may not effectively integrate and take advantage of the semantic anatomical information provided for the lesion detection task in this context.

**Table 3:** Summary of object detection metrics for all experimental setups, with results stratified according to the specific annotation protocols of Annotator 1 (*n* = 16) and Annotator 2 (*n* = 30).

Experiment	FROC@0.1	FROC@0.5	AP@0.1	AP@0.5	mAP@0.1–0.5
nnDetection [baseline] [1]	**0.305**	0.100	0.320	0.084	0.178
nnDetection [baseline] [2]	0.291	**0.118**	**0.471**	**0.131**	**0.345**
nnDetection + TS [1]	0.297	0.061	0.327	0.056	0.167
nnDetection + TS [2]	0.369*	0.145*	0.570*	0.165*	0.409*
Swin RetinaUNeTR [1]	**0.283**	**0.087**	0.305	0.063	0.185
Swin RetinaUNeTR [2]	0.236	0.069	**0.412**	**0.070**	**0.269**
Swin RetinaUNeTR + TS [1]	0.257	0.056	0.268	0.047	0.133
Swin RetinaUNeTR + TS [2]	0.257	**0.081**	**0.443**	**0.096**	**0.300**

Top scores for each metric are presented in bold.

## 4 Discussion

In this study, we investigated the effect of adding anatomical priors for identifying lymphoma lesions in whole-body PET/CT scans. For this, we incorporated anatomical information into the training pipeline using TotalSegmentator to generate segmentation masks of 104 organs from the CT modality. The masks were then provided as additional input channels in conjunction with the PET and CT volumes. Using the nnDetection framework as a baseline, we extended the Retina U-Net architecture by explicitly incorporating anatomical information as an additional input during training. Moreover, we adapted the Swin Transformer architecture for lesion detection and assessed the effect of including extra anatomical information in that model.

**Figure 8:**
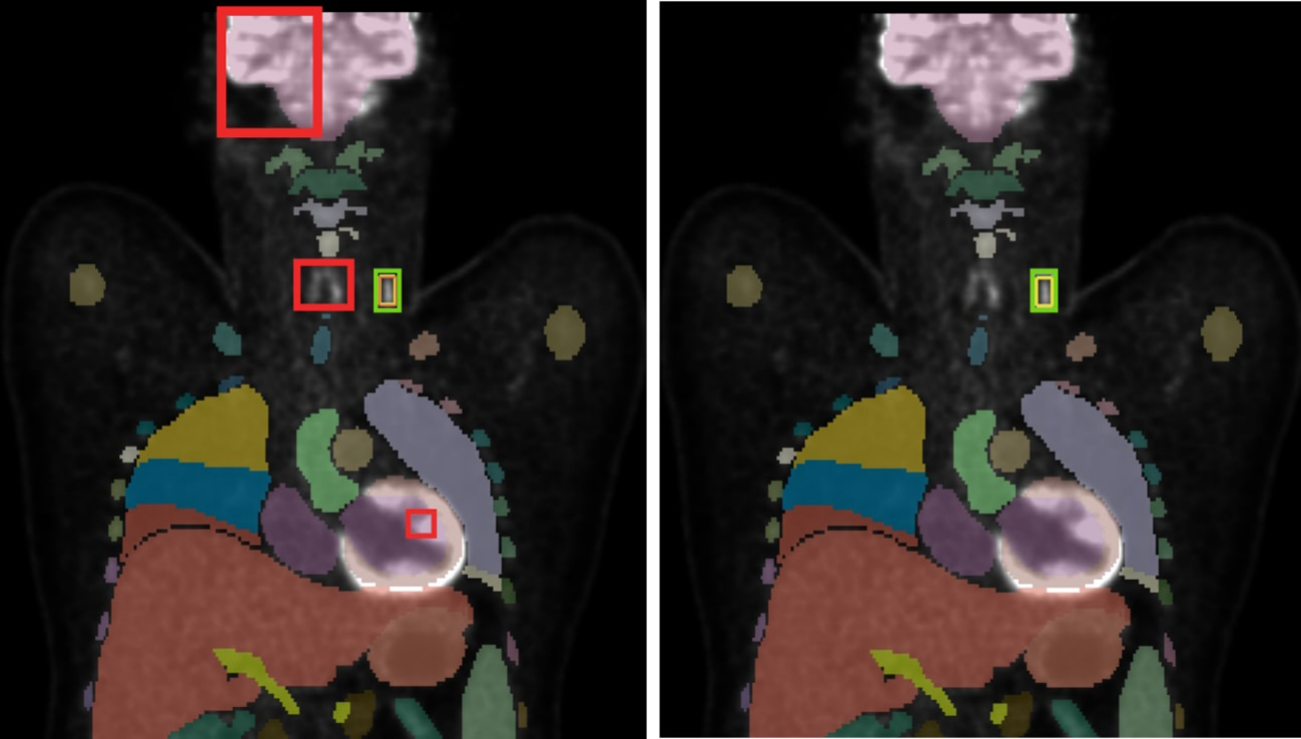
Visualization of detection results: baseline nnDetection (left) vs. enhanced nnDetection (right) with Total Segmentator mask overlay. The incorporation of anatomical information in the enhanced model demonstrates a reduction in false positive detections, particularly evident in the brain, thyroid, and heart regions.

Experimental results demonstrated that the inclusion of anatomical information led to improved performance, particularly in the nnDetection framework. These findings suggest that the network is capable of learning contextual anatomical information, correlating the occurrence of lymphoma lesions with specific organs, while also learning to ignore physiologically high-uptake regions in PET, such as the liver, thyroid, brain, and bladder, as shown in **Figure 8**. This performance boost is likely attributable to the regionally localized nature of convolutional filters used in nnDetection, which appear to benefit from the explicit anatomical context provided. While the absolute performance metrics underscore that the model is not yet suitable for fully autonomous diagnosis, the substantial relative improvements gained through anatomical grounding suggest that such a system could already function as an assistance tool. By highlighting candidate regions that warrant closer inspection, the model serves to reduce the workload on radiologists, even if further refinement is required to reach high-sensitivity clinical thresholds.

**Figure 9:**
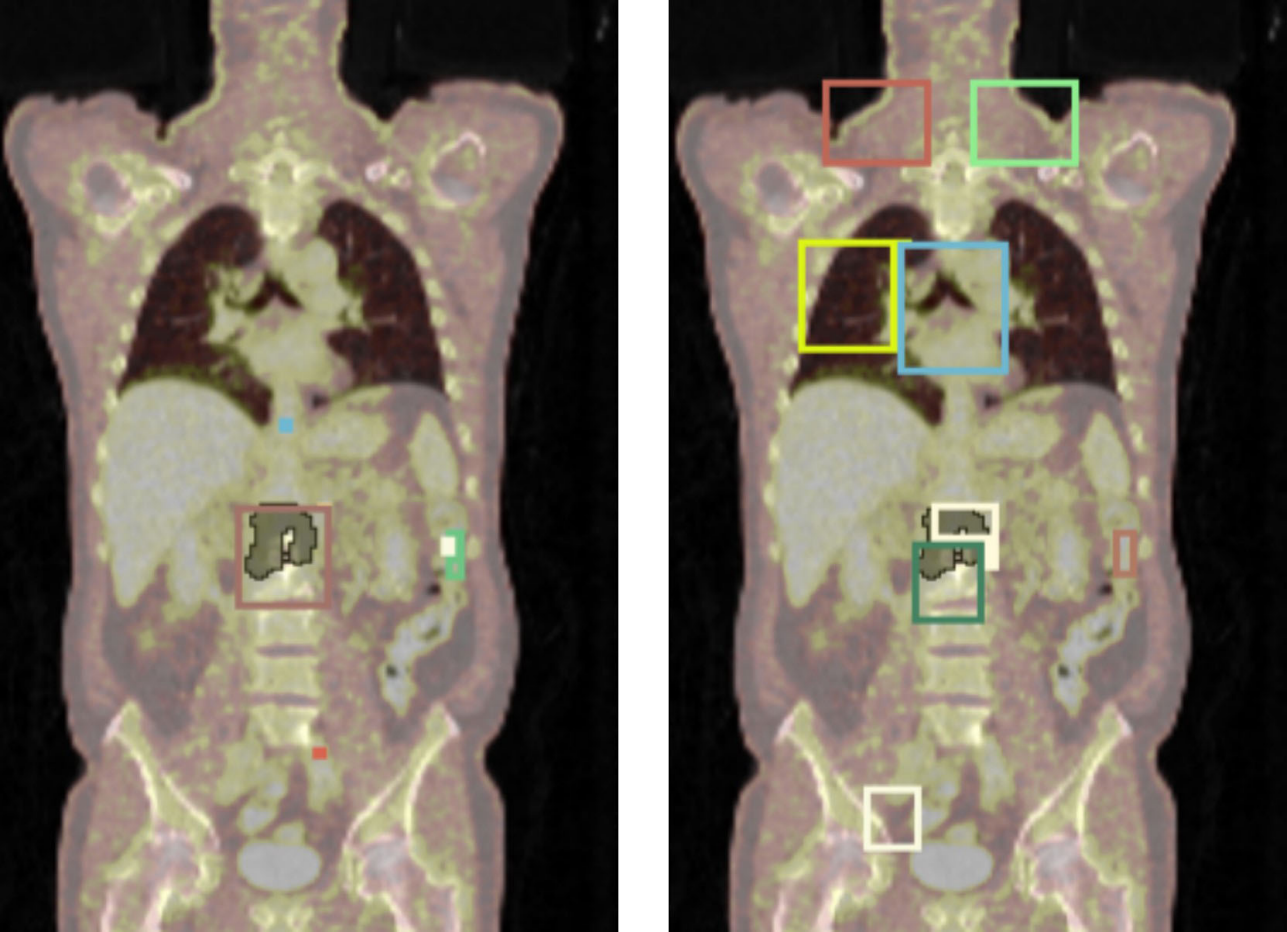
Visualization of detection results in one case with a single lesion in the abdomen. We show the 90th percentile of predicted bounding boxes from RetinaUNet (left) versus Swin RetinaUneTR (right). While RetinaUNet accurately localizes the lesion region with few false positives, Swin RetinaUneTR fails to maintain local focus, generating predictions not only in the lesion but also across the entire body, which impacts the final classification performance.

In contrast, the same benefits were not observed in the Swin Transformer-based architecture. The addition of anatomical input did not lead to significant performance improvements, possibly due to the global attention mechanism inherent in the Swin Transformer. This characteristic may already allow the model to extract contextual dependencies, reducing the marginal gain from the added anatomical information. Furthermore, the hypothesis that a global attention mechanism might dilute or lose fine-grained local information, critical for the detection of small objects, demands further investigation in future studies. Such research could assess whether the adoption of transformer-based architectures inherently limits task learning in segmentation and object detection scenarios where local, high-resolution features are fundamental for identifying small lesions. To provide further context regarding this point, **Figure 9** illustrates the top 10% of predicted bounding boxes for a representative case. A comparison between nnDetection and the Swin-based architecture demonstrates that while nnDetection accurately localizes the tumor region, the Swin-based model exhibits a more global activation pattern, failing to focus effectively on the specific lesion.

When directly comparing the Swin Transformer-based network with the well-established nnDetection framework, it is evident that the former does not yet match the state-of-the-art performance of the latter. This observation highlights two important conclusions: 1) nnDetection continues to demonstrate robust, high-performing capabilities across diverse tasks and remains a strong benchmark in medical image object detection; and 2) the Swin RetinaUNeTR architecture may require further refinement, particularly in the tuning and optimization of the Swin Transformer component, to realize its full potential and achieve competitive performance. The observed performance improvements in CNN-based architectures indicate that anatomy-aware models can better discriminate pathological uptake from normal physiological activity. From a clinical perspective, this has direct implications for reducing false-positive findings, which are a major barrier to the adoption of computer-aided detection systems in PET/CT. A lower false-positive burden could translate into reduced radiologist review time, increased trust in automated prescreening tools, and improved consistency in lesion identification, particularly in high-volume clinical settings or longitudinal follow-up studies.

Our future work will focus on this refinement process and extend the evaluation of the proposed approach to additional medical object detection tasks beyond lymphoma, such as multifocal tumors in the liver or brain, and across different imaging modalities. Moreover, proposing better approaches for warming up Swin Transformers may help to close the current gap between CNN-based and ViT-based lymphoma detection architectures. Since nnDetection is optimized for CNN architectures, it is relevant to propose alternative autoconfiguration mechanisms that can be more effective with ViT architectures at a reasonable cost. In this context, future studies might also explore other transformer-based object detection frameworks, such as DEtection TRansformer (DeTR) (42) variants, to evaluate whether their bipartite matching and end-to-end set prediction logic can better handle the sparsity of medical lesions. However, as demonstrated in (43), DETR-based frameworks still require a hierarchical, multiscale architecture, similar to the Swin Transformer, to successfully detect the small, multifocal, and multiscale objects typically found in medical imaging. Once transformer-based architectures achieve state-of-the-art performance and comparable robustness, the focus can shift toward multi-objective studies. For example, a single network could be leveraged in a multitasking framework to both detect lymphoma lesions and simultaneously classify disease subtypes, such as indolent versus aggressive lymphoma. Finally, as presented in this study, ACCT, which is acquired in standard procedures during PET for correction of photon attenuation of fat and other tissues, can be considered a feasible reference CT modality for future studies. This approach can eliminate the need for an additional contrast-enhanced diagnostic CT acquisition. However, further validation through comparative analyses with and without contrast-enhanced diagnostic CT remains necessary.

Furthermore, future research will address the statistical robustness of our results by transitioning from a single train–validation split to a k-fold cross-validation framework. This will provide more reliable performance estimates and ensure that the gains observed with the Swin Transformer are consistent across various data distributions. Alongside this more rigorous evaluation, we plan to explore alternative pretraining strategies, such as self-supervised, multitask, or domain-specific schemes, to further enhance representation learning. Combining these improved pretraining paradigms with a robust validation strategy will be essential for closing the performance gap between CNN and ViT-based architectures in clinical detection tasks.

It is important to note that one of the primary objectives in the development of deep learning methods is to demonstrate tangible clinical benefits. Currently, we have not studied the influence of adding anatomical priors on reporting time, staging accuracy, or treatment decisions. It is also important to stress that this work focused exclusively on a single disease, and therefore, the results cannot be assumed to generalize to other cancer types without further investigation.

## 5 Conclusion

This work addresses a clinically relevant challenge in whole-body PET/CT imaging, namely, the reliable and automated detection of lymphoma lesions in the presence of complex and heterogeneous physiological uptake patterns. Accurate lesion detection is critical for disease staging, therapy response assessment, and longitudinal follow-up and remains a time-consuming and cognitively demanding task in routine clinical practice.

In this study, we demonstrated that integrating explicit anatomical priors, derived from TotalSegmentator predictions, can significantly improve the performance of CNN-based lesion detection architectures. By providing organ-level contextual information alongside PET and CT inputs, the proposed approach enables the network to better discriminate pathological uptake from normal physiological activity, thereby reducing false-positive detections. Such improvements are particularly relevant for clinical adoption, as false positives represent a major limitation of current computer-aided detection systems and directly impact radiologist trust and workflow efficiency.

In contrast, the incorporation of anatomical information did not yield comparable performance gains in the Swin Transformer-based architecture. This finding suggests that transformer models may already capture global contextual information through their attention mechanisms, or that additional architectural refinement and optimization are required to fully leverage explicit anatomical priors. As a result, further methodological developments, including improved pretraining strategies and architecture-specific tuning, are necessary to close the performance gap between transformer-based and state-of-the-art CNN-based detection frameworks.

Overall, our findings highlight the value of anatomy-aware modeling for medical object detection, particularly in CNN-based approaches, and provide important insights into the current limitations and future potential of vision transformer architectures in PET/CT lesion detection. With continued refinement and broader validation, anatomy-informed deep learning models hold promise for supporting more efficient, consistent, and clinically meaningful decision-making in oncologic imaging.

## Data Availability

Publicly available datasets were analyzed in this study. This data can be found here: https://www.cancerimagingarchive.net/collection/fdg-pet-ct-lesions/.
